# Genomic Islands Identified in Highly Resistant *Serratia* sp. HRI: A Pathway to Discover New Disinfectant Resistance Elements

**DOI:** 10.3390/microorganisms11020515

**Published:** 2023-02-17

**Authors:** Samantha J. McCarlie, Charlotte E. Boucher, Robert R. Bragg

**Affiliations:** Department of Microbiology and Biochemistry, University of the Free State, Bloemfontein 9301, South Africa

**Keywords:** antimicrobial resistance, mobile genetic elements, multidrug efflux pumps, biocide resistance

## Abstract

Molecular insights into the mechanisms of resistance to disinfectants are severely limited, together with the roles of various mobile genetic elements. Genomic islands are a well-characterised molecular resistance element in antibiotic resistance, but it is unknown whether genomic islands play a role in disinfectant resistance. Through whole-genome sequencing and the bioinformatic analysis of *Serratia* sp. HRI, an isolate with high disinfectant resistance capabilities, nine resistance islands were predicted and annotated within the genome. Resistance genes active against several antimicrobials were annotated in these islands, most of which are multidrug efflux pumps belonging to the MFS, ABC and DMT efflux families. Antibiotic resistance islands containing genes encoding for multidrug resistance proteins ErmB (macrolide and erythromycin resistance) and biclomycin were also found. A metal fitness island harbouring 13 resistance and response genes to copper, silver, lead, cadmium, zinc, and mercury was identified. In the search for disinfectant resistance islands, two genomic islands were identified to harbour *smr* genes, notorious for conferring disinfectant resistance. This suggests that genomic islands are capable of conferring disinfectant resistance, a phenomenon that has not yet been observed in the study of biocide resistance and tolerance.

## 1. Introduction

The COVID-19 pandemic has highlighted our need for effective disinfectants, antiseptics, and sanitisers (biocides). The antibiotic resistance crisis can be seen as a warning or foreshadowing of an equally alarming phenomenon of microbial resistance to disinfectants. This means it is troubling that, within the food and agricultural industries and medical environments, resistance to disinfectants amongst microorganisms is emerging at a startling rate [1,2,3,4].

Mobile genetic elements (MGEs) play a significant role in the transfer of genes which confer antimicrobial resistance [5,6,7,8]. Their mobility is brought about by horizontal gene transfer, resulting in populations with reduced susceptibility to various antimicrobials [5,8]. Resistance can develop against several antimicrobials simultaneously, without prior exposure [9]. Genomic island (GI) is an umbrella term for mobile genetic elements found on the bacterial chromosome that have been acquired through horizontal gene transfer, usually between 10 and 200 kb in length [6,10,11]. This overarching term also includes integrated plasmids, integrons, prophages, conjugative transposons, and integrative conjugative elements [6,10,11,12]. These MGEs are then given more specific identities based on their mechanism of transfer (conjugation, transduction, or transformation) and genes present (transposases, integrases etc.) [6,12].

Genomic islands can be further characterised based on the phenotype they confer. For example, pathogenicity islands encode genes that confer an advantage in pathogenicity [13], resistance islands encode antimicrobial resistance genes [14], and metabolic islands contain genes that confer an additive metabolic advantage [6,10].

The bioinformatic identification of genomic islands is achieved using two approaches. The first is via sequence composition, and the second is via comparative genomics [10,11]. Both techniques have respective advantages and limitations, and therefore, a combination of the two provides the most sensitive and precise output [10,12]. IslandViewer4 is the gold standard for genomic island prediction, as it incorporates four different genomic island prediction methods, IslandPick, IslandPath-DIMOB, SIGI-HMM, and Islander [15].

Genomic islands have been found to play a role in antibiotic resistance [8,16]. However, minimal research has been carried out on the role of genomic islands in disinfectant resistance. As this is an emerging issue, more insight into the molecular mechanisms of resistance to disinfectants and other biocides is needed. A genomic island in *Listeria monocytogenes* isolates was found to be responsible for food-borne outbreaks harbouring multiple resistance genes, including an efflux pump involved in benzalkonium chloride resistance (ErmE) [17,18]. Jiang and co-workers (2020) found that the *sug* operon on the bacterial chromosome encoding SMR efflux pumps conferred resistance to benzalkonium chloride. This research brings forth the idea that resistance islands may be the latest genetic element capable of conferring resistance to disinfectants.

Resistance islands are often harboured in multidrug-resistant bacteria as one of many mechanisms to increase survivability [19]. One of these bacteria, *Serratia* sp. HRI, has high disinfectant resistance capabilities and provides a unique opportunity to study resistance to disinfectants and other biocides [20]. Several mechanisms of resistance to disinfectants have been elucidated, with efflux pumps being the most common. However, molecular-based resistance has mostly been limited to the study of plasmids. Little is known about which other mobile genetic elements can play a significant role in the development and dissemination of the disinfectant resistance phenotype. In the search for novel mechanisms of disinfectant resistance, genomic islands and the hypothetical proteins they harbour are attractive targets in the search for novel, previously undescribed mechanisms of resistance. If the molecular basis of disinfectant resistance is better understood, this will help to safeguard our current disinfectants and ensure proper biosafety in the agricultural, food, and medical industries. The aim of this work is to use prediction software and bioinformatic analysis to determine whether genomic islands can contribute to disinfectant resistance. The finding of several resistance islands harbouring known disinfectant resistance genes within this highly resistant isolate suggests that genomic islands can be characterised as a molecular element capable of conferring disinfectant and biocide tolerance and resistance. This paper adds to the evidence that genomic islands are capable of conferring biocide tolerance and resistance.

## 2. Materials and Methods

*Serratia* sp. HRI was isolated from a bottle of Didecyldimethylammonium chloride (DDAC)-based disinfectant [20]. Upon analysis, high levels of resistance to Quaternary Ammonium Compound (QAC) disinfectants were found via Minimal Inhibitory Concentration (MIC) tests [20].

The unusually high level of resistance observed in this isolate, together with its isolation from a bottle of disinfectant, prompted research into this microorganism. The genome of *Serratia* sp. HRI was sequenced and previously published [20]. The raw reads from this sequencing run, described previously, were then assembled again using the PATRIC (v. July 2021) de novo Genome Assembly service with default parameters unless otherwise specified (available at https://www.bv-brc.org/app/Assembly2) [21].

This assembled genome is 5 533 130 bp long, with GC content of 59.1%, an N50 score of 348 770, an L50 of 5, 47 contigs, and 126 RNAs, deposited on NCBI under Genbank Accession No. CP083690.1. This genome was uploaded to IslandViewer4 [15] with *Serratia marcescens* strain N4-5 chromosome sequence as a reference. IslandViewer4 uses four genomic island prediction methods (IslandPick, IslandPath-DIMOB, SIGI-HMM, and Islander) to identify genomic islands [15]. Thereafter, resistance genes are identified by IslandViewer4 using the Resistance Gene Identifier (RGI) from the Comprehensive Antibiotic Resistance Database (CARD) [22], as well as virulence factors from the Virulence Factor Database (VFDB) [23], PATRIC [24], and Victor’s virulence factors (http://www.phidias.us/victors/ (accessed on 11 January 2022)), in addition to 18 919 pathogen-associated genes [25,26]. For further analysis and annotation, the sequence of each genomic island was uploaded to RAST and the NCBI Prokaryotic Genome Annotation Pipeline (PGAP) NCBI annotation tool for additional annotation [27,28].

In the GIs of interest (GI 11, 20, and 76), any gene annotated as a hypothetical or uncharacterised protein was finally run through the PSI-BLAST program [29] and annotated further if any significant hits were found.

## 3. Results

**Figure 1:**
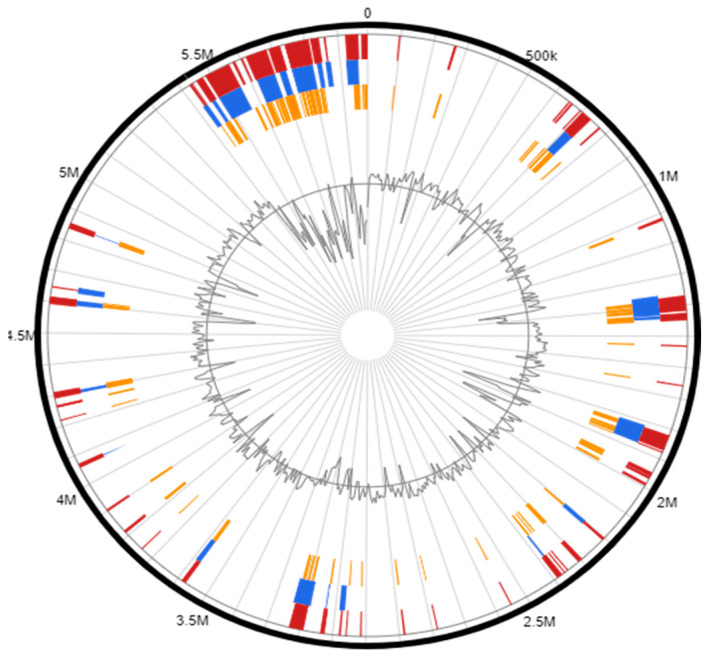
Circular map generated by IslandViewer4 depicting the location of genomic islands within the genome of *Serratia* sp. HRI. Orange bars represent GIs identified via the SIGI-HMM genomic island prediction software, blue bars are GIs identified via IslandPath-DIMOB program, and the integrated GIs identified via all programs used are represented by red bars. Adapted from IslandViewer4 [15].

IslandViewer4 identified 92 genomic islands within the genome of *Serratia* sp. HRI, as depicted in Figure 1. Of the 92 genomic islands, 9 contained known antimicrobial resistance genes or genes implicated in antimicrobial resistance; these genomic islands were predicted via at least two prediction methods. Table 1, Table 2, Table 3 and Table 4 represent the structure of these genomic islands and annotated gene lists [27,30,31]. 

Three of the nine genomic islands are shown in more detail as they contain resistance genes of particular interest (Table 2, Table 3 and Table 4); the remaining six islands are depicted in more detail in the Supplementary section (Tables S1–S6). Resistance island 11 is studied closely due to the number of resistance genes and their combination with hypothetical proteins, transcriptional regulators, and toxin–antitoxin systems. Resistance islands 20 and 76 are of interest as they contain known disinfectant resistance genes and a number of hypothetical proteins.

Genomic island 11 is represented in Table 2. This resistance island contains 78 annotated genes, including 7 genes encoding various efflux pumps. Of the seven genes, these include two copies of permeases of the drug/metabolite transporter (DMT) superfamily and a probable Co/Zn/Cd efflux system membrane fusion protein. Various components of efflux systems, such as an inner-membrane proton/drug antiporter (MSF type) of a tripartite multidrug efflux system, an outer membrane factor (OMF) lipoprotein, and two ABC-type antimicrobial peptide transport system proteins, make up the permease component and ATPase component. There are about 40 hypothetical proteins and multiple transcriptional regulators within this genomic island, including those of the Trx, AcrR, LuxR, and LysR families.

Genes of interest in genomic island 20, represented in Table 3, include a small multidrug resistance efflux protein (SMR), an ABC transporter permease protein, and a probable Co/Zn/Cd efflux system membrane fusion protein. Several genes are associated with conjugative transfer, mobile element proteins, an integron-associated gene, and transposase-associated genes. Hypothetical protein 19 was further annotated by NCBI PGAP as an SMR family transporter, a well-known disinfectant resistance gene.

Genomic island 76, depicted in Table 4, contains 47 genes, including an *smr* gene and an ABC-type multidrug transport system gene, together with a complete toxin–antitoxin system (YoeB/YefM). This genomic island is also a mosaic of several mobile element associated genes, such as an integrase, repeat regions, recombinase, and multiple mobilisation proteins (MobA, MobC). Hypothetical protein 7 in GI 76 had a significant similarity hit in the BLAST program with a multidrug efflux ABC transporter permease/ATP-binding subunit SmdA (Max score: 25.0, Total score: 25.0, Query cover: 74%, E value: 1.9, Per. Ident: 26.51%). This protein is located next to a component of an ABC-type multidrug transport system and is likely part of an efflux system. Hypothetical protein 11, located adjacent to an SMR disinfectant resistance protein, had the highest similarity hit with GNAT family N-acetyltransferase (*Serratia marcescens*) when run through the BLAST program. This family of proteins is responsible for resistance to aminoglycoside antibiotics [32] and could play a role in the antimicrobial resistance of *Serratia* sp. HRI.

Although the following genomic islands were not highlighted, each has interesting characteristics and contains at least one antimicrobial resistance gene. Genomic island 18, depicted in Table S1 in the Supplementary section, contains heavy metal response genes to molybdenum and two ABC-type efflux pump permease components, YbhS and YbhR. These proteins, together with YbhF, form YbhFSR, which functions in tetracycline efflux and Na+(Li+)/H+ transport [33]. Adjacent to these genes is ybhL, a closely related gene whose function is unknown but is hypothesised to be involved in stress response and cell protection by unknown mechanisms [34].

Table S2 represents genomic island 23, which is one of the smallest GIs identified with only four genes. Some argue it should not be identified as a GI due to its small size [11]. However, as it contains a multidrug resistance gene from the DMT superfamily, it is noteworthy.

Genomic island 28, depicted in Table S3, contains genes encoding antibiotic multidrug resistance protein ErmB (macrolide and erythromycin resistance) and an adjacent ABC efflux gene [35,36]. This GI also contains multiple transposase genes and components from insertion sequence element IS911, suggesting this insertion sequence may have played a role in the evolution of this resistance island.

Genomic island 33 is a small island with only one annotated protein, shown in Table S4. The protein annotated is an HtpX protease, which, together with ClpA, is involved in aminoglycoside resistance in *Stenotrophomonas maltophilia* [37,38]. Although this island does not contain the ClpA gene, the HtpX protease has been co-selected with multiple hypothetical proteins, which may aid in its function and could be candidates for further study.

Genomic island 42 is a highly conserved metal response island, described in Table S5, harbouring 13 genes involved in metal response with three complete toxin–antitoxin systems. Multiple toxin–antitoxin systems and several MGE-associated genes suggest this genomic island is mobile and highly conserved within a population. The toxin–antitoxin system, HigA/HigB, has been found to play a regulatory role in virulence and biofilm formation in *Pseudomonas aeruginosa* [39,40]. The metal response genes include those for silver and copper, which are being promoted as used in some products an alternatives to current antimicrobials [41]. These characteristics threaten the efficacy of the potential of this alternative treatment.

A bicyclomycin resistance protein can be found on genomic island 46 in Table S6. This resistance protein, together with error-prone repair (UmuD) and error-prone DNA polymerase (UmuC), could introduce mutations and aid in the evolution of antimicrobial resistance.

## 4. Discussion

Resistance islands are a well-known molecular element capable of conferring antibiotic resistance [42], but little research has been carried out on whether these mobile elements play a role in disinfectant and biocide resistance. Improved sequencing technology and more accessible bioinformatic programs have opened the door to the study of these elements and their impact on the resistance profile. This work aims to use these advances in sequencing technology to identify regions likely characterised as resistance islands contributing to the high levels of disinfectant resistance observed in this isolate.

These results are integrated images and gene annotations generated by the IslandViewer4, RAST, PGAP, and PSI-BLAST programs. A total of 92 genomic islands were found within the genome of *Serratia* sp. HRI, and a few are highlighted here as they are of extrachromosomal origin, identified within a highly resistant microorganism, and harbour antimicrobial resistance genes. The vast amount of genomic islands identified within *Serratia* sp. HRI aligns with the predicted high level of plasticity within the *Serratia* genus [5]. High genomic plasticity can lead to a mosaic of MGEs and can be attributable to resultant antimicrobial resistance [8]. Iguchi and co-workers (2014) found high genome plasticity in a clinical *Serratia marcescens* isolate. Compared to a non-resistant isolate, a mosaic of mobile genetic elements and acquired resistance genes contributed to the high levels of antimicrobial resistance in the clinical isolate [5].

Genomic island 11 was the first presented here and can be described as an all-round resistance and fitness island, as it harbours several annotated resistance genes applicable to various antimicrobials. This genomic island includes partial efflux systems from the MFS, OMF, and ABC families and two copies of complete systems from the DMT efflux family. Efflux genes that are not labelled as resistance genes are also highlighted, as they are part of the genome of a highly resistant isolate, placed within a resistance island, and close to a resistance efflux system. Therefore, they are of interest for further study. This genomic island also carries genes involved in metal response, colicin immunity, transcriptional regulators, and multiple MGE components (insertion sequences, phage integrase, and mobility genes). All four transcriptional regulator families found within this GI have been shown to improve bacterial fitness and survivability. LysR-type transcriptional regulators have been reported to play a role in antibiotic resistance in *Aeromonas* sp. [43]. LuxR transcriptional regulators are involved in biofilm formation and stress response in *Pseudomonas* and *Mycobacterium* sp. [44,45]. AcrR transcriptional regulators and their mutations have been seen to contribute towards drug resistance in *Salmonella* sp. [46]. Finally, the possible regulatory protein thioredoxin (Trx) protects against oxidative stress, a well-established response after treatment by antimicrobials such as disinfectants [47]. Interestingly, more than half of all the genes present in this island are uncharacterised and are listed as hypothetical proteins. As this is a large genomic island and requires metabolic resources to maintain and transcribe these elements, it is intriguing that these genes have not been lost. This suggests that some of these hypothetical proteins which form the majority of this genomic island may have a function and are attractive candidates in the search for novel resistance genes and even novel mechanisms of resistance.

Genomic island 20 contains the first gene directly implicated in disinfectant resistance, the *smr* gene [19,48], as well as an ABC efflux permease protein. This island also contains a metal response gene and multiple conjugative transfer proteins alluding to the origin of this GI. Within this sequence, a mosaic of MGEs, including genes encoding transposases, an integrase, and mobile element proteins, were discovered. Multiple transcription regulators associated with antimicrobial resistance are again present in this GI, including regulators from the LysR family and Tetr families, linked to tetracycline resistance [49,50]. Within this resistance island, 11 out of 41 genes are uncharacterised and annotated as hypothetical proteins. This island contains multiple MGEs, suggesting high plasticity, and the probability of incorporating additional resistance determinants is high.

Genomic island 76 contains a complete toxin–antitoxin system (Yoe-B/YefM), an ABC multidrug efflux-encoding gene and, importantly an *smr* gene. This resistance island is conservable in a population due to the toxin–antitoxin system, and almost two-thirds of the genes in this island are uncharacterised. Out of the 47 genes making up this GI, 29 are hypothetical proteins that have been co-selected and maintained with the antimicrobial resistance genes in this island. These uncharacterised flanking sequences are potential targets in the search for new mechanisms of resistance.

When considered all together, these genomic islands contain multiple antimicrobial resistance genes harboured simultaneously within the genome of *Serratia* sp. HRI, which can confer a wide range of resistance within this single isolate. Although there were many incomplete efflux systems (GIs 11, 18, 19, 20, 28, and 76), bioinformatics and annotation software still have a way to go, and in the years to come, these systems may be annotated differently.

In a field such as disinfectant resistance, where knowledge of mechanisms is minimal, the vast numbers of hypothetical proteins within these resistance islands are attractive targets in searching for novel resistance genes and mechanisms of disinfectant resistance.

It is also interesting that very few genes identified in these islands were assigned to subsystems after annotation. This adds to the notion that bioinformatics and annotation programs need improvement, as more information is needed on where these genes fit into the bacterial metabolism and their function(s).

The plasticity and adaptability of the *Serratia* genome shows the capability of the this genus in acquiring MGEs that can contribute to the decreased susceptibility often observed in the *Serratia* genus [5]. The result is observed in isolates such as *Serratia* sp. HRI, whose genome is an assortment of fitness determinants gathered over time, increasing survivability to a wide range of antimicrobials. To confirm the phenotypic impact of these resistance islands and the extent of their impact, further work will be required.

## 5. Conclusions

There is limited information on whether genomic islands are capable of conferring resistance to disinfectants. Therefore, the genomic islands of *Serratia* sp. HRI will add to the knowledge of antimicrobial resistance and reinforce the idea that genomics islands can be described as the latest molecular element capable of conferring disinfectant resistance. This work also adds to the evidence for the cross-resistance and co-selection of antimicrobial resistance genes within a single organism. This work represents how predictive bioinformatic technology can lead targeted research into antimicrobial resistance. However, this is a starting point and only tells scientists where to look instead of providing a definitive answer. Phenotypic analysis needs to be coupled with predictive software to fully elucidate resistance mechanisms.

The increased use of disinfectants during the COVID-19 pandemic will inevitably give rise to less susceptible populations at an advanced rate. Amidst the pandemic, we are silently and unknowingly selecting disinfectant-resistant microorganisms. By getting ahead of disinfectant resistance, we will be able to safeguard our current disinfectants and ensure infection control in both the agricultural and medical industries.

## Figures and Tables

**Table 1:** Summary of the properties of resistance islands of *Serratia* sp. HRI, including a selection of genes within the resistance islands identified by IslandViewer4.

Genomic Island	Antimicrobial Resistance Genes	Hypothetical Proteins	Toxin-Antitoxin Systems	Mobility Genes	Non-Resistance Efflux Genes	Transcriptional Regulators
11	7	40	2 *	9	0	5
18	2	0	0	0	1	0
20	3	10	0	11	1	3
23	1	1	0	0	0	0
28	1	1	0	3	1	0
33	1	5	0	0	0	0
42	13	23	7 *	13	0	0
46	1	5	0	1	0	0
76	3	28	2	5	0	1

* 1 partial toxin–antitoxin system.

**Table 2:** Gene list of resistance island 11 of *Serratia* sp. HRI (1 370 193 bp–1 419 319 bp, GC content 49.2, size 49 126) identified by IslandViewer4. Gene function was annotated via RAST; any hypothetical or uncharacterised proteins were further analysed via NCBI PGAP and BLAST. Annotated drug resistance genes are highlighted in bold.

	Function	Start	Stop	Length (bp)	Annotation
1	Periplasmic fimbrial chaperone StfD	3	764	762	
2	Hypothetical protein	799	1455	657	Fimbrial protein (*Serratia*)
3	Hypothetical protein	1472	1966	495	Fimbrial protein (*Serratia marcescens*)
4	MrfF	1983	2474	492	
5	Minor fimbrial subunit StfG	2484	3014	531	
6	Hypothetical protein	3158	3697	540	LuxR C-terminal-related transcriptional regulator (*Serratia marcescens*)
7	Hypothetical protein	3715	3888	174	
8	IS1 protein InsB	4211	3969	243	
**9**	**Inner-membrane proton/drug antiporter (MSF type) of tripartite multidrug efflux system**	**6496**	**4208**	**2289**	
10	Transcriptional regulator, LysR family	6637	7539	903	
11	Colicin immunity protein PA0984	7645	8010	366	
12	YpjF toxin protein	8619	8251	369	
13	Uncharacterized protein YagB	9016	8678	339	
14	UPF0758 family protein	9526	9047	480	DNA repair protein RadC (*Serratia marcescens*)
15	Hypothetical protein	9541	9765	225	
16	Hypothetical protein	9887	10,069	183	
17	FIG01222608: hypothetical protein	10,562	10,206	357	
18	Hypothetical protein	11,008	10,697	312	
19	Hypothetical protein	11,323	11,021	303	
20	Hypothetical protein	11,845	11,342	504	
21	Hypothetical protein	12,570	11,842	729	WYL-domain-containing protein *(Serratia marcescens*)
22	Hypothetical protein	13,008	12,772	237	
23	Hypothetical protein	13,903	13,019	885	
24	Hypothetical protein	14,462	15,091	630	Inovirus Gp2 family protein (*Serratia marcescens*)
25	Hypothetical protein	15,213	15,425	213	AlpA family phage regulatory protein *(Serratia marcescens)*
26	Hypothetical protein	15,474	15,632	159	
27	Hypothetical protein	17,366	15,774	1593	DUF3987-domain-containing protein (*Serratia marcescens*)
28	Hypothetical protein	17,395	17,535	141	
29	Hypothetical protein	17,784	17,963	180	ShlB/FhaC/HecB family hemolysin secretion/activation protein (unclassified *Serratia)*
30	Hypothetical protein	17,960	18,208	249	
31	Phosphoglycerate mutase (EC 5.4.2.11)	18,243	18,860	618	
32	Il-IS_2, transposase	19,280	18,843	438	
33	Hypothetical protein	20,125	19,277	849	SMP-30/gluconolactonase/LRE family protein (*Serratia marcescens*)
34	Oxidoreductase, short-chain dehydrogenase/reductase family	20,988	20,122	867	
35	Transcriptional regulator, LysR family	21,133	21,426	294	
36	Mobile element protein	22,121	21,606	516	
37	Insertion element IS401 (*Burkholderia multivorans*) transposase	22,400	22,173	228	
38	Phage integrase	22,837	22,553	285	
39	Phage-associated DNA N-6-adenine methyltransferase	23236	22,955	282	
40	Hypothetical protein	23,677	23,531	147	
41	Hypothetical protein	23,838	23,680	159	
42	Hypothetical protein	23,837	23,971	135	
43	Hypothetical protein	24,125	23,997	129	
44	FIG01055438: hypothetical protein	24,208	24,387	180	
45	Hypothetical protein	24,456	24,620	165	
46	Hypothetical protein	24,617	24,712	96	
47	Hypothetical protein	24,706	24,834	129	
48	Hypothetical protein	25,094	24,936	159	
**49**	**Efflux transport system, outer membrane factor (OMF) lipoprotein**	**25,470**	**26,885**	**1416**	
**50**	**ABC-type antimicrobial peptide transport system, permease component**	**26,885**	**28,021**	**1137**	
**51**	**ABC-type antimicrobial peptide transport system, ATPase component**	**28,039**	**28,764**	**726**	
**52**	**Probable Co/Zn/Cd efflux system membrane fusion protein**	**28,775**	**29,683**	**909**	
53	2-hydroxy-3-keto-5-methylthiopentenyl-1-phosphate phosphatase related protein	29,715	30,416	702	
54	Hydrolase, alpha/beta fold family	30,413	31,303	891	
**55**	**Permease of the drug/metabolite transporter (DMT) superfamily**	**31,300**	**31,659**	**360**	
**56**	**Permease of the drug/metabolite transporter (DMT) superfamily**	**31,662**	**32,087**	**426**	
57	Hypothetical protein	33,118	32,228	891	
58	FIG110192: hypothetical protein	34,184	33,120	1065	Peptidogalycan biosysnthesis protein (*Serratia*)
59	Aminotransferase, class III	35,560	34184	1377	
60	Mobile element protein	35,743	35,856	114	
61	Hypothetical protein	36,927	35,869	1059	ATP-binding protein (*Serratia* sp. HRI)
62	Two-component transcriptional response regulator, LuxR family	37,624	36,929	696	
63	Hypothetical protein	37,940	38,161	222	
64	Core lipopolysaccharide phosphoethanolamine transferase EptC	38,236	39,933	1698	
65	Two-component response regulator	40,672	40,502	171	
66	Two-component response regulator	40,948	40,685	264	
67	Hypothetical protein	41,166	41,032	135	
68	Hypothetical protein	42,468	41,395	1074	RelA/SpoT-domain-containing protein (*Serratia*)
69	Hypothetical protein	42,751	42,542	210	
70	Hypothetical protein	42,965	42,822	144	
71	Hydrolase, alpha/beta fold family	43,881	43,006	876	
72	Monooxygenase, flavin-binding family	45,404	43,878	1527	
73	Transcriptional regulator, AcrR family	46,310	45,717	594	
74	Hypothetical protein	46,429	46,310	120	
75	Hypothetical protein	46,428	46,628	201	
76	MmcH	46,648	47,535	888	
77	Hypothetical protein	47,657	47,857	201	
78	Possible regulatory protein Trx	47,870	49,126	1257	

**Table 3:** Gene lists of genomic island 20 of *Serratia* sp. HRI (1 822 085 bp-1 869 515 bp, GC content 52.4, size 47 430 bp) identified via IslandViewer4. Gene function was annotated via RAST; any hypothetical or uncharacterised proteins were further analysed via NCBI PGAP and BLAST.

	Function	Start	Stop	Length (bp)	Annotation
1	Conjugative transfer protein TrbK	326	3	324	
2	Conjugative transfer protein TrbJ	1082	339	744	
3	Conjugative transfer protein TrbE	3529	1079	2451	
4	Conjugative transfer protein TrbD	3811	3542	270	
5	Conjugative transfer protein TrbC	4194	3808	387	
6	Conjugative transfer protein TrbB	5261	4191	1071	
7	CopG-domain-containing protein	5734	5258	477	
8	Coupling protein VirD4, ATPase required for T-DNA transfer	7728	5731	1998	
9	Transcriptional regulator, LysR family	8034	8939	906	
10	Hypothetical protein	9221	9751	531	
11	Transposase and inactivated derivatives	9796	10,032	237	
**12**	**Small multidrug resistance family (SMR) protein**	**10,578**	**10,261**	**318**	
13	Probable lipoprotein	10,900	10,637	264	
14	Transcriptional regulator, LysR family	11,838	10,933	906	
15	Hypothetical protein	13,335	11,932	1404	TolC family protein
16	Transcriptional regulator, TetR family	13,446	14,087	642	
**17**	**Probable Co/Zn/Cd efflux system membrane fusion protein**	**14,084**	**15,250**	**1167**	**MULTISPECIES: efflux RND transporter periplasmic adaptor subunit**
18	Hypothetical protein	15,275	18,379	3105	MULTISPECIES: efflux RND transporter permease subunit
19	Hypothetical protein	18,460	18,807	348	MULTISPECIES: SMR family transporter
20	Hypothetical protein	18,823	19,443	621	
**21**	**ABC transporter, permease protein (cluster 9, phospholipid)**	**19,440**	**20,597**	**1158**	
22	Mobile element protein	21,909	21,205	705	
23	Integron integrase IntI1	21,900	22,196	297	
24	Mobile element protein	22,571	23,209	639	
25	Transposase	23,176	26,100	2925	
26	Beta-glucosidase (EC 3.2.1.21)	27,418	26,180	1239	
27	Putative polysaccharide export protein YccZ precursor	27,383	28,471	1089	
28	Tyrosine-protein kinase (EC 2.7.10.2)	28,730	30,892	2163	
29	Hypothetical protein	30,933	32,171	1239	
30	Hypothetical protein	32,197	33,204	1008	
31	Hypothetical protein	33,223	33,972	750	
32	Poly(glycerol-phosphate) alpha-glucosyltransferase (EC 2.4.1.52)	34,315	35,256	942	
33	Hypothetical protein	35,283	36,419	1137	
34	UDP-galactopyranose mutase (EC 5.4.99.9)	36,474	37,625	1152	
35	Low-molecular-weight protein-tyrosine-phosphatase (EC 3.1.3.48) => Etp	38,004	38,438	435	
36	Tyrosine-protein kinase (EC 2.7.10.2)	38,450	40,621	2172	
37	Hypothetical protein	40,702	41,862	1161	
38	Hypothetical protein	41,828	43,288	1461	MULTISPECIES: aldo/keto reductase
39	Glycosyltransferase	43,278	44,186	909	
40	Glycosyl transferase, group 1	44,233	45,276	1044	
41	Glycosyltransferase	45,351	47,300	1950	

**Table 4:** Gene lists of genomic island 76 of *Serratia* sp. HRI (5 688 450 bp-5 725 416 bp, GC content: 44.0, Size: 36 966 bp) identified via IslandViewer4. Gene function was annotated via RAST; any hypothetical or uncharacterised proteins were further analysed via NCBI PGAP and BLAST.

	Function	Start	Stop	Length (bp)	Annotation
1	Hypothetical protein	923	411	513	Hypothetical protein (*Serratia* sp. SSNIH1)
2	Polyketide synthase modules and related proteins	4124	1122	3003	
3	Hypothetical protein	4338	4222	117	
4	Autoinducer synthase	4424	5584	1161	
5	Hypothetical protein	5859	6110	252	
**6**	**ABC-type multidrug transport system, permease component**	**6668**	**6546**	**123**	
7	Hypothetical protein	6969	6658	312	Multidrug efflux ABC transporter permease/ATP-binding subunit SmdA (*Serratia marcescens*) (WP_033641139.1)
8	Hypothetical protein	7032	8279	1248	MbeB family mobilization protein (*Serratia marcescens*)
9	MobA	8378	8599	222	
**10**	**Small multidrug resistance family (SMR) protein**	**8666**	**8998**	**333**	
11	Hypothetical protein	9165	8995	171	GNAT family N-acetyltransferase (*Serratia marcescens*)
12	Hypothetical protein	9377	9207	171	
13	Hypothetical protein	9746	9531	216	
14	Mobilization protein MobC	10,181	10,339	159	
15	Hypothetical protein	11,258	10,875	384	
16	Hypothetical protein	11,371	12,447	1077	
17	Hypothetical protein	13,804	12,512	1293	Site-specific integrase (*Serratia*)
18	Probable site-specific recombinase	15,011	13,806	1206	
19	Transcriptional regulator, AlpA-like	15,550	15,344	207	
20	Hypothetical protein	16,511	15,651	861	DUF6387 family protein (*Serratia*)
21	Hypothetical protein	16,691	16,575	117	
22	Hypothetical protein	17,617	16,709	909	DUF4760-domain-containing protein (Enterobacterales)
23	Hypothetical protein	17,972	17,856	117	
24	Hypothetical protein	18,388	19,452	1065	
25	Repeat region	19,395	19,521	127	
26	Replication protein	20,789	19,809	981	
27	Hypothetical protein	21,202	20,993	210	
28	Hypothetical protein	21,229	21,357	129	Conjugal transfer protein TraD (*Yersinia*)
29	Hypothetical protein	21,836	21,384	453	
30	Mobilization protein	21,871	23,106	1236	
31	Hypothetical protein	23,121	23,711	591	tRNA modification GTPase (*Yersinia enterocolitica*)
32	Restriction enzyme BcgI alpha chain-like protein (EC:2.1.1.72)	23,769	25,805	2037	
33	Hypothetical protein	25,847	26,941	1095	
34	YoeB toxin protein	27,235	26,981	255	
35	YefM protein (antitoxin to YoeB)	27,483	27,232	252	
36	Hypothetical protein	27,667	28,959	1293	
37	Repeat region	27,757	27,883	127	
38	Phage integrase	28,952	29,149	198	
39	Type I restriction-modification system, restriction subunit R (EC 3.1.21.3)	29,715	30,176	462	
40	Hypothetical protein	30,943	30,173	771	MFS transporter (*Serratia*)
41	Hypothetical protein	31,191	31,382	192	GNAT family N-acetyltransferase (*Paenibacillus xylanexedens*)
42	Hypothetical protein	31,502	31,410	93	Phytanoyl-CoA dioxygenase family protein (*Serratia*)
43	Hypothetical protein	31,702	32,502	801	
44	Nodulation protein nolO (EC 2.1.3.-)	32,512	34,344	1833	
45	Hypothetical protein	34,355	34,492	138	
46	Hypothetical protein	34,496	35,602	1107	G-D-S-L family lipolytic protein (*Serratia*)
47	Hypothetical protein	35,662	36,966	1305	ATP-grasp-domain-containing protein (*Serratia*)

## Data Availability

Sequence data used in this article have been deposited with the DDBJ/EMBL/GenBank Data Libraries under Genbank Accession No. CP083690.1.

## Supplementary Materials

The following supporting information can be downloaded at: https://www.mdpi.com/article/10.3390/microorganisms11020515/s1, Table S1: Gene lists of genomic island 18 of *Serratia* sp. HRI (1 655 571 bp–1 660 471 bp, GC content 62.3, Size 4 900 bp) identified via IslandViewer4 and annotated via RAST, Table S2: Gene lists of genomic island 23 of *Serratia* sp. HRI (1 875 362 bp–1 879 853 bp, GC content: 45.1, Size 4 491 bp) identified via IslandViewer4 and annotated via RAST, Table S3: Gene lists of genomic island 28 of *Serratia* sp. HRI (2 294 061 bp–2 309 315 bp, GC content: 48.1, Size: 15 254 bp) identified via IslandViewer4 and additional annotated via RAST, Table S4: Gene lists of genomic island 33 of *Serratia* sp. HRI (2 548 843 bp–2 553 244 bp, GC content 41.9, Size: 4 401 bp) identified via IslandViewer4 and annotated via RAST, Table S5: Gene lists of genomic island 42 of *Serratia* sp. HRI (3 188 478 bp–3 232 330 bp, GC content: 51.2, Size: 43 852 bp) identified via IslandViewer4 and annotated via RAST, Table S6: Gene lists of genomic island 46 of *Serratia* sp. HRI (3 571 957 bp–3 586 537 bp, GC content: 51.7, Size: 14 580) identified via IslandViewer4 and annotated via RAST.
