# Supplementary material for: Genomic Islands Identified in Highly Resistant Serratia sp. HRI: A Pathway to Discover New Disinfectant Resistance Elements
Source: Microorganisms. 2023 Feb 17;11(2):515. doi: 10.3390/microorganisms11020515 (PMC9964261; doi:10.3390/microorganisms11020515)
Supplement: Supplementary file 1 [file microorganisms-11-00515-s001.zip › microorganisms-2189307-supplementary.pdf]

Supplementary Materials

Table S1. Gene lists of genomic island 18 of *Serratia* sp. HRI (1 655 571bp - 1 660 471bp, GC content 62.3, Size 4 900 bp) identified by IslandViewer4 and annotated by RAST

	Function	Start	Stop	Length (bp)
1	**ABC-type efflux pump permease component YbhS**	**3**	**1154**	**1152**
2	**ABC-type efflux pump permease component YbhR**	**1167**	**2273**	**1107**
3	Inner membrane protein YbhL	3059	2349	711
4	Molybdopterin synthase catalytic subunit MoaE (EC 2.8.1.12)	3657	3190	468
5	Molybdopterin synthase sulfur carrier subunit	3905	3660	246
6	Cyclic pyranopterin monophosphate synthase (EC 4.6.1.17)	4381	3902	480
7	Molybdenum cofactor biosynthesis protein MoaB	4900	4385	516

Genomic island 18 contains 2 confirmed ABC-type efflux pump permease components YbhS and YbhR, as well as an inner membrane protein YbhL.

Table S2. Gene lists of genomic island 23 of *Serratia* sp. HRI (1 875 362 bp -1 879 853bp, GC content: 45.1, Size 4 491 bp) identified by IslandViewer4 and annotated by RAST

	Function	Start	Stop	Length (bp)
1	**Permease of the drug/metabolite transporter (DMT) superfamily**	**1256**	**3**	**1254**
2	hypothetical protein	2715	1249	1467
3	Glycosyl transferase, group 2 family protein	3562	2708	855
4	dTDP-rhamnosyl transferase RfbF (EC 2.-.-.-)	4491	3559	933

Genomic island 23 is one of the smallest islands identified and contains a permease of the drug/metabolite transporter (DMT) superfamily.

Table S3. Gene lists of genomic island 28 of *Serratia* sp. HRI (2 294 061bp - 2 309 315bp, GC content: 48.1, Size: 15 254 bp) identified by IslandViewer4 and additional annotated by RAST

	Function	Start	Stop	Length (bp)
1	Similar to citrate lyase beta chain, 3	869	3	867
2	Siderophore synthetase superfamily, group B	1041	2900	1860
3	Ferrichrome-iron receptor	2929	5040	2112
4	Orn/DAP/Arg family decarboxylase	5052	6452	1401
5	Pyridoxal-5'-phosphate-dependent enzyme beta superfamily (fold type II)	6464	7477	1014

6	Tauropine dehydrogenase	7477	8634	1158
**7**	**Multidrug resistance protein ErmB**	**8637**	**9863**	**1227**
**8**	**ABC transporter, substrate-binding protein (cluster 8, B12/iron complex)**	**9860**	**11020**	**1161**
9	probable class-V aminotransferase	10989	12173	1185
10	hypothetical protein	12181	12597	417
11	Transposase InsN for insertion sequence element IS911	13027	13272	246
12	Transposase InsO for insertion sequence element IS911	13458	13856	399
13	Transposase	14178	15254	1077

Genomic island 28 contains the multidrug resistance protein ErmB conferring resistance to macrolide and erythromycin antibiotics, as well as an ABC transporter, substrate-binding protein. There are also multiple transposase genes within this genomic island and multiple iron-associated genes.

Table S4. Gene lists of genomic island 33 of *Serratia* sp. HRI (2 548 843bp – 2 553 244bp, GC content 41.9, Size: 4 401 bp) identified by IslandViewer4 and annotated by RAST

	Function	Start	Stop	Length (bp)
1	hypothetical protein	3	200	198
**2**	**Protease HtpX**	**278**	**1156**	**879**
3	hypothetical protein	1551	1667	117
4	hypothetical protein	1683	1856	174
5	hypothetical protein	2030	3229	1200
6	hypothetical protein	3232	4401	1170

Table S5. Gene lists of genomic island 42 of *Serratia* sp. HRI (3 188 478bp – 3 232 330bp, GC content: 51.2, Size: 43 852 bp) identified by IslandViewer4 and annotated by RAST

	Function	Start	Stop	Length (bp)
1	Mobile element protein	3	464	462
2	Resolvase/integrase	1028	471	558
3	hypothetical protein	1393	1022	372
4	hypothetical protein	2697	1390	1308
5	Mobile element protein	2696	4189	1494
6	Mobile element protein	4197	4319	123
7	Transposase	4357	5055	699
8	hypothetical protein	5602	5474	129
9	Toxin HigB	6101	6430	330
10	Antitoxin HigA	6411	6692	282
11	hypothetical protein	6968	6807	162
12	repeat region	6898	8102	1205
13	Transposase InsH for insertion sequence element IS5	6970	7950	981
14	hypothetical protein	8114	7989	126
15	Bacterial non-heme ferritin (EC 1.16.3.2)	8767	8267	501
16	Mobile element protein	9744	9022	723

Sequence data from this article have been deposited with the DDBJ/EMBL/GenBank Data Libraries under Accession No. NZ_WIXF00000000

17	Transposase InsH for insertion sequence element IS5	9785	10438	654
18	hypothetical protein	10894	11025	132
19	hypothetical protein	11060	11317	258
20	Mobile element protein	12199	11990	210
21	Mobile element protein	12957	12376	582
22	hypothetical protein	13163	13288	126
23	GGDEF domain protein	13485	13315	171
24	repeat region	13447	14651	1205
25	Transposase InsH for insertion sequence element IS5	14615	13599	1017
**26**	**Copper resistance protein CopC**	**15319**	**14939**	**381**
**27**	**Copper resistance protein B**	**16249**	**15359**	**891**
**28**	**Multicopper oxidase**	**18072**	**16255**	**1818**
**29**	**Copper-binding protein PcoE**	**18306**	**18755**	**450**
30	Cell wall endopeptidase, family M23/M37	19044	19781	738
31	hypothetical protein	20012	19815	198
**32**	**Lead, cadmium, zinc and mercury transporting ATPase (EC 3.6.3.3) (EC 3.6.3.5); Copper-translocating P-type ATPase (EC 3.6.3.4)**	**22041**	**20053**	**1989**
33	hypothetical protein	22045	22446	402
**34**	**CopG protein**	**23067**	**22627**	**441**
**35**	**Copper/silver efflux RND transporter, transmembrane protein CusA**	**26300**	**23154**	**3147**
**36**	**Copper/silver efflux RND transporter, membrane fusion protein CusB**	**27603**	**26311**	**1293**
**37**	**Copper/silver efflux RND transporter, periplasmic protein CusF**	**28070**	**27717**	**354**
**38**	**Copper/silver efflux RND transporter, outer membrane protein CusC**	**29484**	**28099**	**1386**
**39**	**Copper-sensing two-component system response regulator CusR**	**29671**	**30354**	**684**
**40**	**Copper sensory histidine kinase CusS**	**30347**	**31822**	**1476**
**41**	**Silver-binding protein silE precursor**	**31956**	**32504**	**549**
42	hypothetical protein	32652	33002	351
43	hypothetical protein	33187	33062	126
44	Hnh endonuclease	34150	33188	963
45	hypothetical protein	34283	34429	147
46	hypothetical protein	34568	34446	123
47	FIG116849: hypothetical protein	35662	34640	1023
48	FIG131328: Predicted ATP-dependent endonuclease of the OLD family	37209	35647	1563
49	VapC toxin protein	37420	37283	138
50	VapC toxin protein	37697	37434	264
51	VapB protein (antitoxin to VapC)	37924	37694	231
52	hypothetical protein	38463	38233	231
53	CcdA protein (antitoxin to CcdB)	38512	38730	219
54	CcdB toxin protein	38732	39037	306

Sequence data from this article have been deposited with the DDBJ/EMBL/GenBank Data Libraries under Accession No. NZ_WIXF00000000

55	hypothetical protein	39095	39406	312
56	hypothetical protein	39456	39791	336
57	hypothetical protein	39825	40841	1017
58	hypothetical protein	40885	41046	162
59	Resolvase	41093	41818	726
60	hypothetical protein	41834	41962	129
61	hypothetical protein	41984	42280	297
62	hypothetical protein	42904	42770	135
63	Mobile element protein	42929	43852	924

Genomic island 42 is one of the larger genomic islands identified consisting of 63 coding sequences. This island contains 13 genes involved in metal response as well as three complete toxin-antitoxin systems including a HigA/HigB system. Mobile genetic elements may have integrated into this genomic island suggested by the presence of multiple mobile element proteins and the presence of transposase, resolvase and integrase genes.

Table S6. Gene lists of genomic island 46 of *Serratia* sp. HRI (3 571 957bp – 3 586 537bp, GC content: 51.7, Size: 14 580) identified by IslandViewer4 and annotated by RAST

	Function	Start	Stop	Length (bp)
1	Polyketide synthase modules and related proteins	6866	3	6864
2	Long-chain-fatty-acid--CoA ligase (EC 6.2.1.3)	7130	7035	96
3	putative exported protein	7319	7152	168
**4**	**Bicyclomycin resistance protein**	**8599**	**7418**	**1182**
5	hypothetical protein	10248	8905	1344
6	hypothetical protein	10389	10835	447
7	hypothetical protein	10832	11392	561
8	hypothetical protein	12778	11603	1176
9	transposase, putative	13451	12960	492
10	Error-prone, lesion bypass DNA polymerase V (UmuC)	13978	13628	351
11	Error-prone repair protein UmuD	14400	13993	408
12	hypothetical protein	14580	14425	156

Genomic island 46 contains a bicyclomycin resistance protein, representing an antibiotic resistance island.

Sequence data from this article have been deposited with the DDBJ/EMBL/GenBank Data Libraries under Accession No. NZ_WIXF00000000
